# Periodontitis-induced systemic inflammation exacerbates atherosclerosis partly via endothelial–mesenchymal transition in mice

**DOI:** 10.1038/s41368-019-0054-1

**Published:** 2019-07-01

**Authors:** Jin Sook Suh, Sol Kim, Kristina I. Boström, Cun-Yu Wang, Reuben H. Kim, No-Hee Park

**Affiliations:** 1 The Shapiro Family Laboratory of Viral Oncology and Aging Research, UCLA School of Dentistry, Los Angeles, CA USA; 2 Department of Medicine, David Geffen School of Medicine at UCLA, Los Angeles, CA USA; 3 Division of Oral Biology and Medicine, UCLA School of Dentistry, Los Angeles, CA USA; 4 UCLA Jonsson Comprehensive Cancer Center, Los Angeles, CA USA; 5 Department of Bioengineering, UCLA Samueli School of Engineering, Los Angeles, CA USA

**Keywords:** Cardiovascular diseases, Periodontitis

## Abstract

Growing evidence suggests close associations between periodontitis and atherosclerosis. To further understand the pathological relationships of these associations, we developed periodontitis with ligature placement around maxillary molars or ligature placement in conjunction with *Porphyromonas gingivalis* lipopolysaccharide injection at the ligature sites (ligature/*P.g*. LPS) in Apolipoprotein E knock out mice and studied the atherogenesis process in these animals. The mice were fed with high fat diet for 11 weeks and sacrificed for analyzing periodontitis, systemic inflammation, and atherosclerosis. Controls did not develop periodontitis or systemic inflammation and had minimal lipid deposition in the aortas, but mice receiving ligature or ligature/*P.g*. LPS showed severe periodontitis, systemic inflammation, and aortic plaque formation. The aortic plaque contained abundant macrophages and cells expressing both endothelial and mesenchymal cell markers. The severity of periodontitis was slightly higher in mice receiving ligature/*P.g*. LPS than ligature alone, and the magnitude of systemic inflammation and aortic plaque formation were also notably greater in the mice with ligature/*P.g*. LPS. These observations indicate that the development of atherosclerosis is due to systemic inflammation caused by severe periodontitis. In vitro, *P.g*. LPS enhanced the secretion of pro-inflammatory cytokines from macrophages and increased the adhesion of monocytes to endothelial cells by upregulating the expression of adhesion molecules from endothelial cells. Moreover, secretory proteins, such as TNF-α, from macrophages induced endothelial–mesenchymal transitions of the endothelial cells. Taken together, systemic inflammation induced by severe periodontitis might exacerbate atherosclerosis via, in part, causing aberrant functions of vascular endothelial cells and the activation of macrophages in mice.

## Introduction

Periodontitis, a chronic local inflammatory disease of the periodontium, is induced by oral microorganisms, primarily Gram-negative bacteria and spirochetes.^1^ These microorganisms generate biofilms by aggregating and adhering to the surface of the teeth and the gingival pockets resulting in the induction of local inflammation. In addition, periodontitis is a systemic inflammatory condition. The patients with periodontitis show higher serum pro-inflammatory cytokines such as tumor necrosis factor (TNF)-α, interleukin (IL)-1β, or IL-6 in comparison to healthy controls.^2^ Thus periodontitis is linked to many systemic diseases, such as rheumatoid arthritis,^3^ aspiration pneumonia,^4^ type 2 diabetes mellitus,^5^ cancer,^6^ and cardiovascular disease (CVD).^7,8^ Among them, CVD has received most attention, as many epidemiological and clinical studies have shown a clear link between periodontitis and CVD.^9–11^ For example, periodontal bacteria such as *Porphyromonas gingivalis*, *Tannerella forsythia*, *or Treponema denticola* have been identified in 52% of atherosclerotic specimens;^12^ treatment of periodontitis has been shown to reduce systemic inflammation and confer favorable effects on atherosclerosis;^7^ and *P. gingivalis* was identified in coronary plaques of patients through a cohort study.^13^ Moreover, oral infection of *P. gingivalis* was reported to accelerate the development of atherosclerosis in apolipoprotein E knock out (*ApoE*^−*/−*^) mice,^14^ and periodontal therapy reduced the systemic and aortic inflammation caused by periodontitis in *ApoE*^−/−^ mice.^15^

Although the detailed mechanisms of the initiation, development, and progression of atherosclerosis remain unclear, recent studies have shown an important role of endothelial-to-mesenchymal transition (EndMT) of aortic endothelial cells in atherogenesis.^16–18^ EndMT contributes to the fibrotic process of atherosclerotic plaque formation which leads to exacerbated stiffness of the perivascular walls and progressive cardiac failure.^19^ In this process, endothelial cells lose expression of endothelial cell-specific proteins and simultaneously show to morphologic changes similar to mesenchymal cells with an expression of mesenchymal cell-specific proteins.^20,21^ Due to the EndMT, the endothelium also loses its integrity as a barrier in blood vessels and allows the extravasation of monocytes and macrophages into the vascular intima.^22,23^ In addition, EndMT-derived mesenchymal-like cells destabilize the atherosclerotic plaques by altering the collagen–matrix metalloproteinase balance.^24^ Therefore, EndMT is viewed as a critical step for the initiation and progression of atherosclerosis.

In the present study, we induced severe periodontitis with ligature placement at maxillary second molars or ligature placement in conjunction with the injection of *P. gingivalis* lipopolysaccharides (Ligature/*P.g*. LPS) in *ApoE*^*−/−*^ mice and investigated the process of atherogenesis in mice, along with in vitro studies. Our study suggests that ligature-induced periodontitis promotes systemic inflammation, which in turn exacerbates atherosclerosis in *ApoE*^*−/−*^ mice possibly by causing aberrant functions of vascular endothelial cells and the activation of macrophages in mice.

## Results

### Ligature placement or ligature placement in conjunction with *P.g.* LPS injection induced periodontitis and increases the level of systemic pro-inflammatory cytokines in mice

**Fig. 1:**
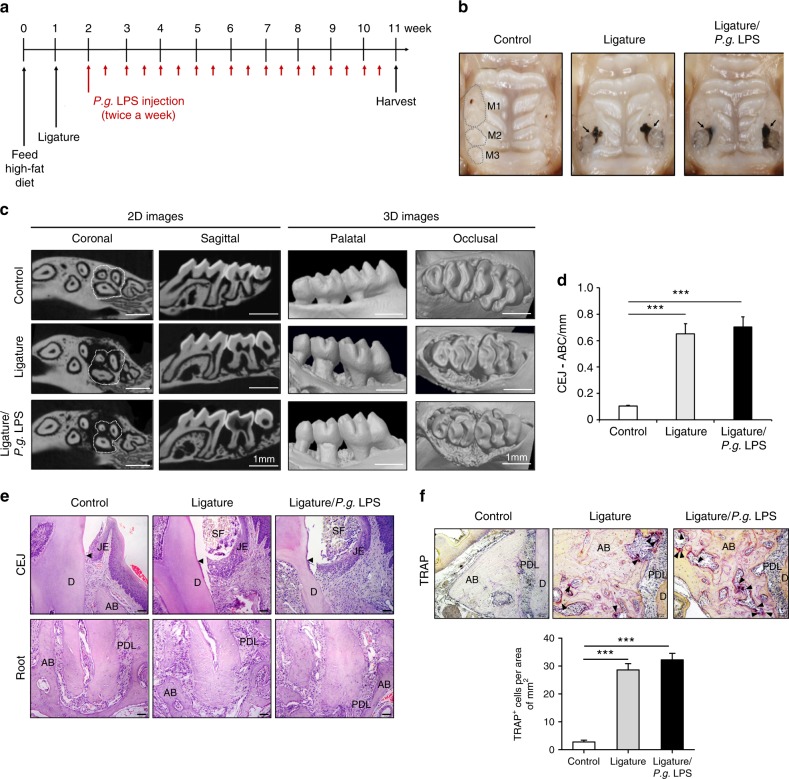
Ligature or Ligature/*P.g*. LPS induced periodontitis in *ApoE*^−*/−*^ mice. **a** Timeline of the study. **b** Palatal tissues including the ligatured upper second maxillary molars at the time of mice sacrifice. Black arrows indicate 6-0 silk suture used for ligature placement. Swelling of palates was noticed. **c** Two-dimensional or three-dimensional μCT images of maxillae in the control mice (*n* = 5), mice receiving ligature (*n* = 5), or ligature/*P.g*. LPS (*n* = 5). Scale bar: 1 mm. **d** Measurement of alveolar bone loss: Distance (mm) from the CEJ to ABC of the second molars. **e** Hematoxylin and Eosin (H&E) staining of the maxillary second molar. Scale bar: 50 μm. **f** TRAP staining for the presence of osteoclasts (pink color; black star) from periodontal tissue. Bone resorption was confirmed by the presence of osteoclasts. TRAP-positive osteoclasts number per area was counted under a blinded manner by two people. Scale bar: 50 μm. Abbreviations: D, dentin; SF, silk fibroin; AB, alveolar bone; JE, junctional epithelium; PDL, periodontal ligament. ***P < 0.001 in one-way ANOVA. Results represent the means ± SD performed in triplicate

**Fig. 2:**
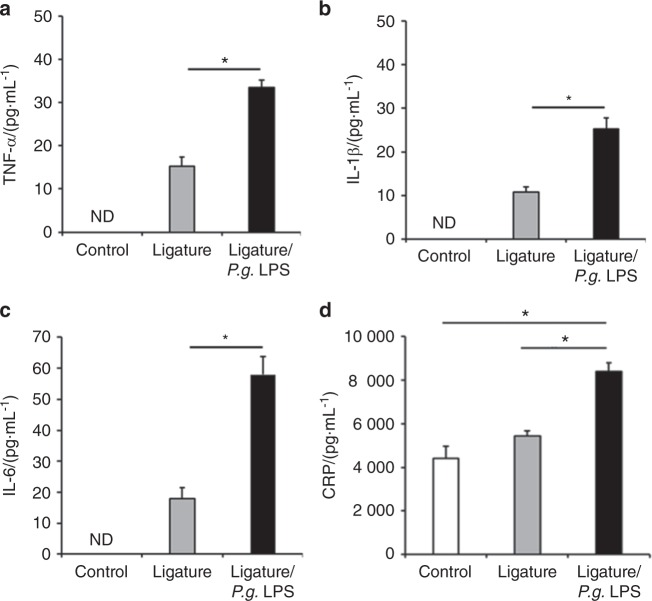
Periodontitis induced by Ligature placement or Ligature/*P.g*. LPS increased serum level of pro-inflammatory cytokines in *ApoE*^*−/*−^ mice. **a**–**d** Levels (pg·mL^−1^) of TNF-α, IL-1β, IL-6, and CRP from the mice sera. They were measured on pre-coated ELISA plates. ND not detected. *P < 0.05 in one-way ANOVA. Results represent the means ± SD performed in triplicate

To investigate the effect of periodontitis on atherogenesis, we induced periodontitis in *ApoE*^*−/−*^ fed a high fat diet (HFD) by placing silk-ligatures around the maxillary second molars. In other group of mice, we combined the placement of ligatures with biweekly injections of *P.g*. LPS, an endotoxin of a major pathogen associated with human periodontitis, into the palatal area of the ligated tooth, since mice are not natural hosts for *P. gingivalis*.^25^ Both groups of mice were compared to control *ApoE*^*−/−*^ mice on HFD. When the mice were harvested at 11 weeks post-HFD nourishing (Fig. 1a), we noted significant palatal swelling around the maxillary second molars with ligature placement or ligature/*P.g*. LPS injection (Fig. 1b). The μCT analysis revealed a severe 7-fold increase of alveolar bone loss in the mice with ligature or ligature/*P.g*. LPS compared to the control mice, as measured by the distance between the cementum–enamel junction (CEJ) and the alveolar bone crest (ABC) (Fig. 1c, d). The amount of bone resorption was slightly higher in the mice with ligature/*P.g*. LPS than in mice ligature alone (Fig. 1d). Histologic examination showed significant alterations in the organization of the junctional epithelium and deep subgingival pockets in periodontitis-induced mice. While the epithelium of the control mice showed typical features of normal murine junctional epithelium, the periodontitis-induced mice showed disorganized architectures including epithelial proliferation into the connective tissues, thickening of periodontal ligaments, and alveolar bone loss (Fig. 1e). TRAP-positive osteoclasts in the alveolar bone were found slightly higher in the mice with ligature/*P.g*. LPS than in mice with ligature alone, but they were not found in that of control mice (Fig. 1f). In mice receiving ligature placement alone, high levels of pro-inflammatory cytokines, such as TNF-α, IL-1β, and IL-6 were detected in the serum, while they were not detected in control mice. The serum levels of these cytokines were further enhanced in mice receiving ligature/*P.g*. LPS (Fig. 2a–c) in comparison to those in mice receiving ligature alone. Similar differences in the increase in the serum C-reactive protein (CRP) level were also observed in the different groups of mice (Fig. 2d).

### Ligature-induced periodontitis increased the aortic lipid deposition and plaque formation in mice

**Fig. 3:**
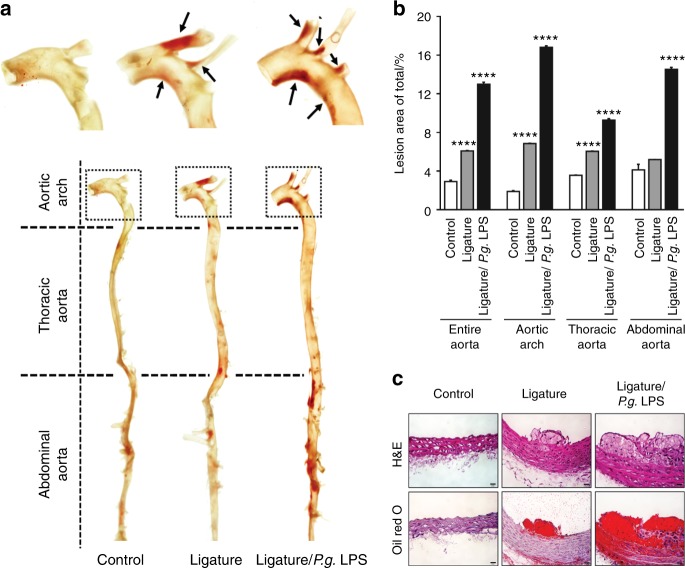
Periodontitis induced by ligature placement or ligature/*P.g*. LPS increased plaque formation in the entire artery. **a** Representative photographs of Oil Red O-stained entire aortas from control (*n* = 5), mice receiving ligature placement (*n* = 5) and mice receiving ligature/*P.g*. LPS (*n* = 5). **b** Quantification of O Red Oil-stained areas at different segment (entire aorta, aortic arch, thoracic region, and abdominal region). **c** Representative photographs of H&E and Oil Red O-stained cross-sections from aortic arches. Scale bar: 50 μm. ****P < 0.000 1 in one-way ANOVA. Results represent the means ± SD performed in triplicate

To find whether the ligature-induced periodontitis induced an exacerbation of atherogenesis, we sacrificed the mice and harvested the aortas after 11 weeks of HFD, a time period that was not expected to cause significant lipid deposition in the aortas of control mice. As expected, we detected negligible amounts of aortic lipid deposition in the HFD-fed *ApoE*^−/−^ control mice, whereas ligature placement and ligature/*P.g*. LPS injection progressively increased the lipid deposition in entire aorta, aortic arch, thoracic aorta, and the abdominal aorta (Fig. 3a, b). Consistent with these observations, histological examination of Oil Red O-stained sections revealed increased atherosclerotic plaque formations in the ligature group, and especially in the ligature/*P.g*. LPS group, as compared to controls (Fig. 3c). Interestingly, no significant differences were detected in serum levels of total cholesterol, triglyceride, high-density lipoprotein-cholesterol (HDL-C), and non-HDL-C (Supplementary Fig. 1A–D), suggesting that the severity of atherosclerosis, rather than high cholesterol levels, is closely linked to the degree of systemic inflammation in this animal model.

**Fig. 4:**
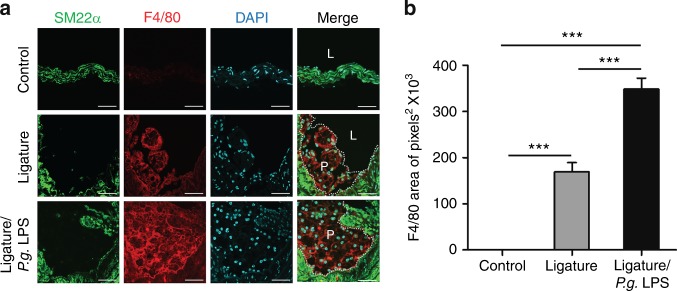
Atherosclerotic lesions exacerbated by periodontitis contain higher number of monocytes and macrophages. **a** Immunofluorescence analysis of atherosclerotic plaques with anti-SM22α (green) and anti-F4/80 (red) antibodies and **b** quantification of F4/80-positive area. Scale bars: 20 μm. Nuclei were stained with DAPI (blue). L lumen, P plaque. ***P < 0.001 in one-way ANOVA

### Atherosclerotic plaques developed by ligature-induced periodontitis contained abundant macrophages and cells with both endothelial and mesenchymal phenotypes

**Fig. 5:**
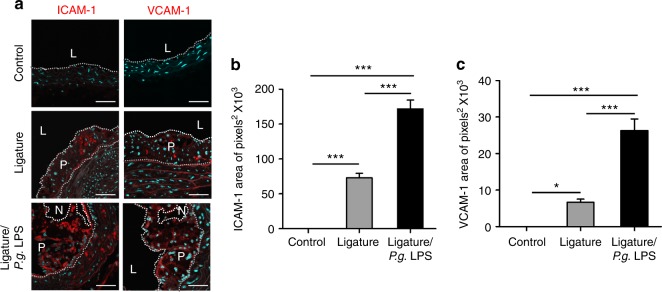
Atherosclerotic lesions exacerbated by periodontitis contain a notable amount of adhesion molecules. **a** Expression of ICAM-1 (red) and VCAM-1 (red) in atherosclerotic plaques and **b** quantification of ICAM-1- or **c** VCAM-1-positive area. *P < 0.05; ***P < 0.001 in one-way ANOVA

**Fig. 6:**
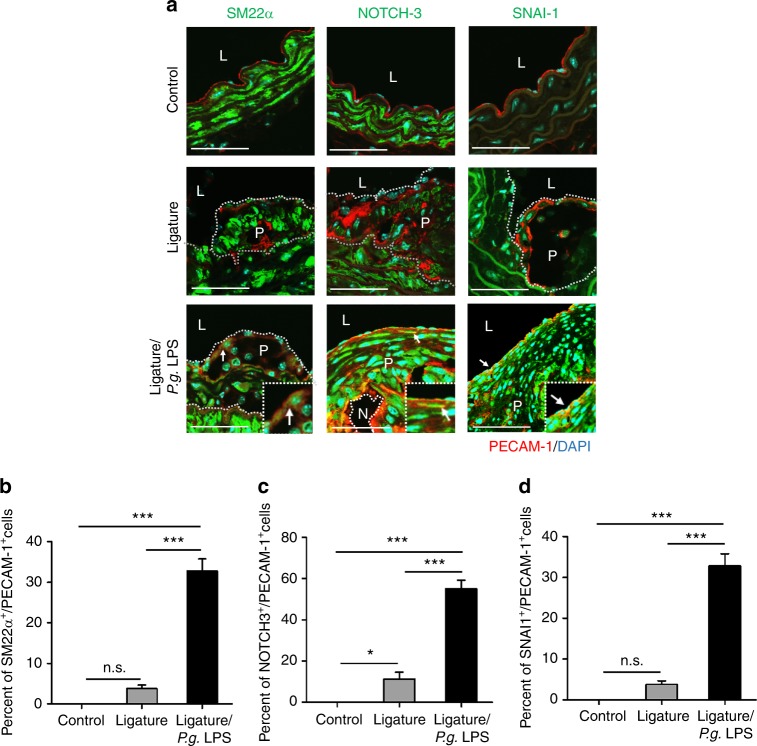
Atherosclerotic lesions exacerbated by periodontitis contain cells expressing both endothelial and mesenchymal protein markers. **a** Immunofluorescence staining of PECAM-1 (red), SM22α (left panel; green), NOTCH-3 (middle panel; green) and SNAI-1 (right panel; green) in the aortic arches of the mice. Enlarged images are shown on the right-bottom side in each image. Scale bars: 20 μm. Nuclei were stained with DAPI (blue). L, lumen; P, plaque; N, necrotic area. White arrows indicate co-expressed cells with both of two signals such as PECAM-1 and SM22α, PECAM-1 and NOTCH-3, or PECAM-1 and SNA-I1. **b**–**d** Quantification of the number of luminal endothelial cells expressing SM22α, NOTCH-3 and SNAI-1. n.s. not significant. *P < 0.05; ***P < 0.001 in one-way ANOVA

To assess the cellular components of the plaques, the plaques were stained with various cell markers for confocal immunofluorescence analysis. The results showed enhanced staining for F4/80, a marker for macrophages, in mice with severe periodontitis with ligature placement or ligature/*P.g*. LPS injections (Fig. 4a, b), indicating an enhanced presence of macrophages in the plaques. The plaques from the mice with periodontitis also showed enhanced staining for adhesion molecules such as intercellular adhesion molecule-1 (ICAM-1) and vascular cell adhesion protein-1 (VCAM-1) (Fig. 5a–c), suggesting the presence of cells with possible endothelial phenotypes in the plaques. In addition, we found co-localization of platelet endothelial cell adhesion molecule-1 (PECAM-1), an endothelial cell adhesion molecule, with EndMT markers such as smooth muscle protein 22 alpha (SM22α) (Fig. 6a, b), neurogenic locus notch homolog protein-3 (NOTCH-3) (Fig. 6a, c), and zinc finger protein SNAI1 (SNAI-1) (Fig. 6a, d), indicating possible EndMT of endothelial cells.

**Fig. 7:**
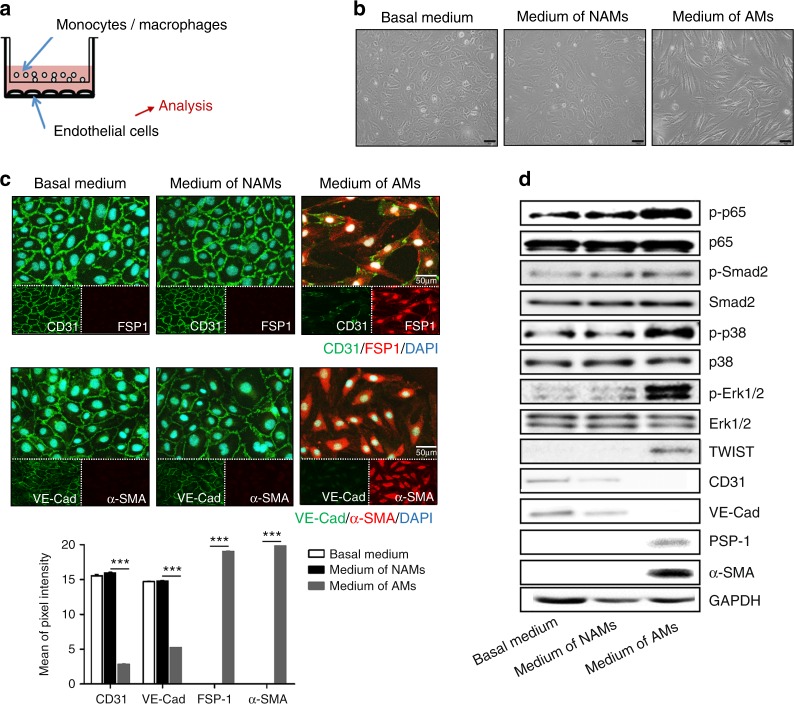
Induction of EndMT of HUVECs by macrophages. **a** Schematic representation in the profile of a co-culture system. THP1 monocytes or THP1-derived activated monocytes were plated in the upper Transwell and, simultaneously, HUVECs were plated on the bottom of the well. **b** Representative phase-contrast microscopic pictures of HUVECs after 20 h co-culture without monocytes or activated monocytes; non-PMA activated monocytes (NAMs) and PMA activated macrophages (AMs) in the inset. Scale bar: 100 μm. **c** Representative immunofluorescent images of the expression of CD31 (green), VE-cad (green), FSP-1 (red) and α-SMA (red) in co-cultured HUVECs and quantification of the expression in the images using ImageJ. Nuclei were counterstained with DAPI (blue). Scale bar: 50 μm. **d** Phosphorylation of EndMT-related signaling molecules such as Smad2, p38, ERK and p65, and protein expressions of CD31, VE-cad, FSP-1 and α-SMA in co-cultured HUVECs. ***P < 0.001 compared with control cells (non-treated cells)

### *P.g.* LPS caused phenotypic changes of human embryonic endothelial cells (HUVECs) in vitro

To lineate the higher exacerbation of atherosclerosis in mice receiving ligature/*P.g*. LPS compared to mice with ligature alone, we investigated the effects of *P.g*. LPS on the phenotypes of vascular endothelial cells. As phenotypic changes of vascular endothelial cells, such as expression of adhesion molecules and EndMT, are critical initial steps and progression of atherosclerosis, we determined the effect of *P.g*. LPS on the expression of adhesion molecules and EndMT from HUVECs in vitro. As expected, we found that the expression of VCAM-1 and ICAM-1 was increased significantly in HUVECs after exposure to *P.g*. LPS (Supplementary Fig. 2). Furthermore, our functional assay showed that the ability to bind calcein-labeled monocytes (THP-1 cells) was significantly higher in HUVECs pre-treated with *P.g*. LPS compared to the control cells (Supplementary Fig. 2G–H). Since the aortic plaques contained cells with increased expression of EndMT markers (Fig. 6a–d), we also investigated whether *P.g*. LPS was able to induce markers of EndMT from HUVECs in vitro. Interestingly, there was no notable change in the morphology of HUVECs in response to *P.g*. LPS and only slight changes in the mRNA and protein expression levels of the endothelial markers, vascular endothelial-cadherin (VE-Cadherin) and CD31, and the mesenchymal marker fibroblast specific protein-1 (FSP-1) (Supplementary Fig. 2A–E).

### Macrophages released pro-inflammatory cytokines and induced phenotypic changes of HUVECs, and exposure of macrophages to *P.g.* LPS augmented the secretion of cytokines from macrophages and macrophage-induced phenotypic alterations of HUVECs

**Fig. 8:**
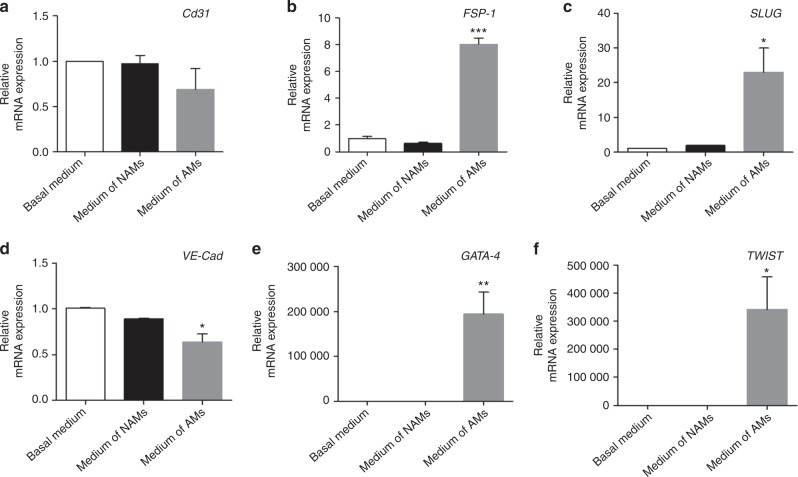
Enhanced expression of EndMT-related genes from HUVECs by macrophages. Relative mRNA expressions of EndMT-related genes in co-cultured HUVECs. Beta-actin served as loading control. *P < 0.05; **P < 0.01; ***P < 0.001 compared control cells (non-treated cells). Results represent the means ± SD performed in triplicate

Inasmuch as there were abundant presence of macrophages and exacerbated EndMT on atherosclerotic plaques in the group receiving ligature/*P.g*. LPS that showed the highest levels of pro-inflammatory cytokines in mouse sera which are known to play a critical role in atherogenesis,^26^ we investigated the effect of macrophages on the expression of cytokines and EndMT markers from HUVECs. We co-cultured macrophages and HUVECs using Transwell®, which allowed macrophages (insert) communicate with endothelial cells (bottom well) via secreted proteins such as cytokines from macrophages (Fig. 7a for schematic diagram of the co-culture system). When HUVECs were cultured with regular media or co-cultured with non-PMA activated monocytes (NAMs), their morphologies were not altered. However, HUVECs co-cultured with PMA-activated macrophages (AMs) underwent phenotypic changes: Morphological transformation of HUVECs to spindle-shaped mesenchymal cells (Fig. 7b); decreased expression of the endothelial cell markers, such as CD31 and VE-Cadherin and increased expression of the mesenchymal cell markers, e.g., FSP-1 and α-SMA (Fig. 7c). Also, phosphorylation of p65, p38, and Erk1/2 was detected as well as expression of TWIST, an EndMT marker in these spindle-shaped cells, without notable Smad2 phosphorylation (Fig. 7d). Moreover, gene expression of other EndMT markers, such as zinc finger protein SNAI2 (SLUG), GATA-binding factor-4 (GATA-4), and twist-related protein (TWIST) was significantly increased in these HUVECs co-cultured with macrophages (Fig. 8a–f). These data strongly suggest that proteins secreted from macrophages induced EndMT of vascular endothelial cells.

**Fig. 9:**
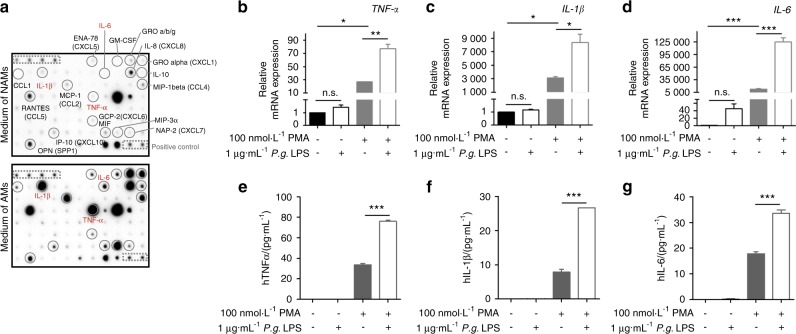
*P.g.* LPS enhanced the expression and secretion of pro-inflammatory cytokines from macrophages. **a** Cytokine array of cytokines and chemokines from the culture media of two different type of phenotypic THP1 cells. Conditioned media from non-PMA activated monocytes (NAMs; monocytes) and 100 nmol·L^−1^ PMA activated monocytes (AMs; macrophages) were evaluated using a human cytokine array. **b**–**d** Relative mRNA expressions of TNF-α, IL-1β and IL-6. **e**–**g** Secreted TNF-α, IL-1β and IL-6 protein levels in the culture media by enzyme-linked immunosorbent assay. *P < 0.05; **P < 0.01; ***P < 0.001 in one-way ANOVA. Results represent the means ± SD performed in triplicate

**Fig. 10:**
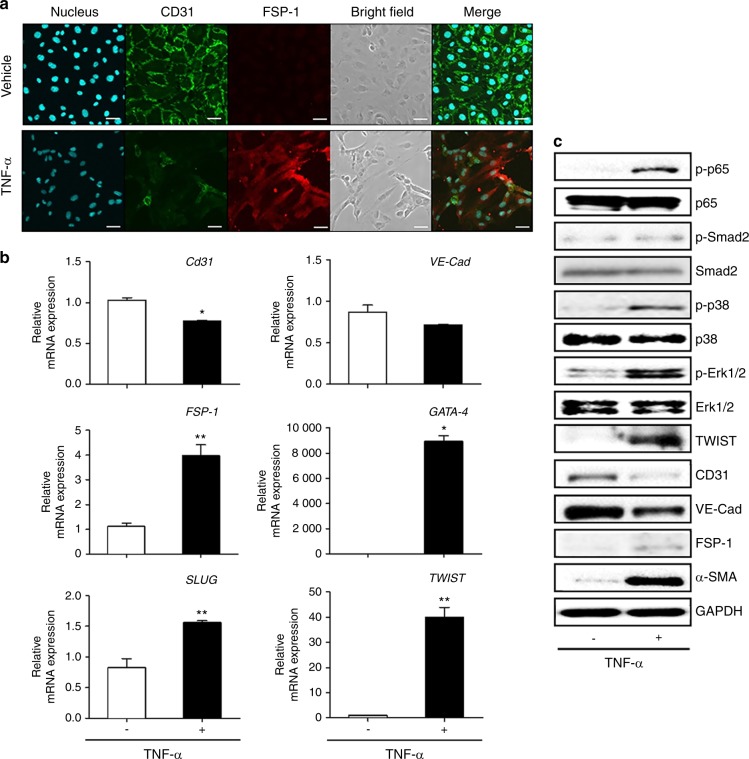
TNF-α induced EndMT by upregulating the expression of EndMT markers in HUVECs. **a** Exposure of HUVECs to TNF-α (10 ng/ml) for 2 days induced morphological changes (bright field) and increased the level of fibroblast-specific protein-1 (FSP-1) in HUVECs. Scale bars: 50 μm. **b** Expression levels of EndMT-related genes determined by qPCR. Beta-actin served as loading control. **c** Representative Western blots of EndMT-related signaling molecules as well as mesenchymal and endothelial proteins. *P < 0.05; **P < 0.01 in one-way ANOVA. Results represent the means ± SD performed in triplicate

In order to detect the nature of the factors in the co-cultured media responsible for the EndMT of vascular endothelial cells, we performed cytokine arrays from the culture media. The arrays showed high levels of pro-inflammatory cytokines, such as TNF-α, IL-1β, and IL-6, in the media co-cultured with macrophages, but not with monocytes (Fig. 9a, Supplementary Tables 1 and 2). Interestingly, such pro-inflammatory cytokines were also detected in the serum of mice with ligature-induced periodontitis, and the level of the cytokines in serum was further enhanced by the injection of *P.g*. LPS in the mice with ligature placement (Fig. 2a–d). Similarly, an exposure of macrophages to *P.g*. LPS in vitro, further enhanced the expression and secretion of TNF-α, IL-1β, and IL-6 from the macrophages (Fig. 9b–g). These data indicate that ligature-induced periodontitis activated macrophages and thereby induced systemic inflammation whose magnitude was further potentiated by local injection of *P.g*. LPS. Moreover, we confirmed that TNF-α, the major pro-inflammatory cytokine released from macrophages induced EndMT of HUVECs in vitro: distinct morphological changes to mesenchymal phenotypes with the loss of CD31 expression and an increase of FSP-1 expression (Fig. 10a–c). Taken together, ligature-induced periodontitis induced systemic inflammation, which subsequently produced vascular inflammation and possible EndMT of vascular endothelial cells, resulting in the formation of aortic plaques.

## Discussion

There are several animal models to recapitulate human periodontal diseases.^27^ Among them, the ligature-induced periodontitis model has not been utilized in mice for periodontal research due to the difficulty in an access to the oral cavity, technical difficulties, and mechanical induction of periodontal inflammation instead of bacterial induction. However, placing ligature around a tooth is known to cause dental plaque accumulation and oral mucosal ulceration, reproducibly inducing periodontitis-like lesions including tissue detachment and bone loss.^27–29^ Therefore, we adopted the ligature-induced periodontitis mouse model to investigate the effect of severe periodontitis on atherogenesis in *ApoE*^*−/*−^ mice, as wild-type mice do not develop atherosclerosis under HFD. It is also known that “the number of lamellae in the normal arterial media layer is small and the characteristic medial *vasa vasora* seen in the large arteries of humans is not observed in mice. Thus, unlike humans, wild-type mice seldom develop atherosclerosis in the coronary arteries.”^30^

Our studies confirmed that severe periodontitis promoted atherosclerosis most likely by inducing systemic inflammation. In the study, we used *P.g*. LPS instead of *P. gingivalis* infection to increase the severity of periodontitis and stimulate the condition of human periodontitis. According to a previous study,^31^ *P. gingivalis* was shown to cause septic shock-like symptoms and even animal death because of causing bacteremia systemically. In contrast, application of *P.g*. LPS instead of *P. gingivalis* could diminish the risk and strongly stimulate inflammatory signals through its receptor, TLR2, because of the advantage of high purity for mechanism analysis.

Unlike *Escherichia coli* LPS, local administration of *P.g*. LPS is known to induce weak systemic inflammation, although it caused intense local inflammation.^32,33^ Therefore, the higher severity of systemic inflammation in mice receiving ligature/*P.g*. LPS than that in mice receiving ligature alone is most likely due to more severe local inflammation with ligature/LPS, although the alveolar bone losses were similar in those two groups (Fig. 2a–d). As the ligature placement on teeth induced periodontitis with severe alveolar bone loss within 3 weeks.^34^ it is possible that ligature/*P.g*. LPS could further induce local inflammation, in addition to the alveolar bone loss, resulting in the induction of more severe systemic inflammation. This possibility deserves further investigation in the future. Indeed, higher aortic lipid deposition and plaque formation in mice receiving ligature/*P.g*. LPS is most likely due to more severe systemic inflammation by *P.g*. LPS than in mice receiving ligature alone, suggesting that the intensity of the atherosclerosis development depends on the magnitude of systemic inflammation caused by severe periodontitis. Our data are in line with previous reports, which indicated that anti-inflammatory therapy targeting IL-1β innate immunity pathway, such as Canakinumab, notably reduced the recurrent CVD.^35,36^

Emerging evidence suggests that activated endothelial cells can transit to ectopic cell type such as fibroblasts and calcifying cells through EndMT.^16^ In this process, endothelial cells lose their specific endothelial marker proteins such as PECAM-1 and VE-Cadherin while simultaneously acquiring mesenchymal marker proteins including α-SMA, FSP-1, and NOTCH-3, and manifesting migratory, invasive, and proliferative phenotypes.^20,21^ In the present study, microscopic findings of the atherosclerotic plaques developed in mice with ligature-induced periodontitis showed that the plaques contained abundant macrophages and cells expressing both endothelial and mesenchymal cell markers, indicating the possible presence of EndMT (Figs. 4–6). The exact mechanisms of EndMT remain unknown, but many in vitro studies have demonstrated that pro-inflammatory cytokines or endotoxins induces EndMT via Smad, Erk/MEK (extracellular signal-regulated kinase), PI3K (phosphoinositide 3-kinase), or p38 MAPK (mitogen-activated protein kinase) signaling pathways.^37–39^ In our study, we found that p38, Erk1/2 and p65, but not Smad2, signaling pathways might be associated with the EndMT conversion of HUVECs in vitro. The result was in line with invariable TGF-β1 and 2 levels, which well-known major signals to activate Smad signaling pathway, in the supernatant from AMs compared with that from NAMs (Supplementary Table 2, lines F4 and F8). Consistent with immunoblotting results of TNF-α treated HUVECs, p65 phosphorylation known as a major inflammatory transcription factor was also increased in the HUVECs cultured with AMs (Fig. 10). The results of the present study provide interesting insights into the signaling mechanisms that mediate EndMT, which warrants further investigation.

Aberrant activation of vascular endothelial cells by vascular inflammation is a critical initiation step in atherogenesis. The process includes expression of adhesion molecules from endothelial cells, adhesion of monocytes to endothelial cells, EndMT of vascular endothelial cells, and the penetration of endothelial cells undergoing EndMT into the arterial intima. In addition, it allows penetration of oxidized lipid and various immune cells freely into the intima stimulating the formation of plaques.^40^ As periodontitis increased the serum level of pro-inflammatory cytokines, such as TNF-α, IL-1β, and IL-6, which could be released from residential or circulatory macrophages in mice with periodontitis, we confirmed that cytokines released from macrophages induced EndMT in HUVECs. Our study also supports a previous study indicating that activation of macrophages and EndMT of endothelial cells are associated with atherogenesis.^41^ Moreover, the presence of adhesion molecules such as VCAM-1 and ICAM-1 in atherosclerotic plaques suggests that the endothelial cells may have undergone alterations that allows for increased binding capacity of other cells including monocytes and macrophages. We also observed co-localization of PECAM-1, endothelial cell adhesion molecule with EndMT markers, such as NOTCH-3 or SNAI-1. These data again suggest that macrophage recruitment and EndMT conversion of endothelial cells might contribute to the atherogenesis.

In response to *P.g*. LPS, there was no notable change in the morphology of HUVECs and only slight changes in the mRNA and protein expression levels of the endothelial markers, VE-Cadherin and CD31, and the mesenchymal marker, FSP-1 (Supplementary Fig. 2A–E). These data suggest that *P.g*. LPS might not be able to directly induce EndMT in HUVECs. Thus, we believe that the presence of cells stained with EndMT markers might be due to inflammatory cytokines released from macrophages in mice with periodontitis as shown in the following section (Fig. 7c). Also, *P.g*. LPS might indirectly induce EndMT by enhancing the expression and secretion of various pro-inflammatory cytokines from macrophages as shown in Fig. 9b–g.

It is worthwhile to note a previous study in which *P. gingivalis* accelerates atheroma formation by shifting the lipid profile.^42^ In contrast, our study showed non-significant changes in the serum lipid levels (Supplementary Fig. 1). This difference between Maekawa’s study and ours may be attributed to the different delivery methods; whole *P. gingivalis* vs. *P.g. LPS*, mouth inoculation vs. gingival tissue injection, and absent of ligature vs. presence of ligature. Nonetheless, our model was sufficient to induce atherosclerosis development (Fig. 3), suggesting that changes in lipid level alone may be required but not sufficient to exacerbate atherosclerosis. On the other hand, our study demonstrated that increased systemic inflammation was directly associated with atherosclerosis development in mice (Figs. 1–3), suggesting the importance of chronic systemic inflammation in the pathophysiology of atherosclerosis.

In summary, we demonstrated in this study that periodontitis exacerbated atherogenesis and that the development of atherosclerosis was dependent on the severity of systemic inflammation generated by periodontitis, but not the severity of periodontal tissue destruction *per se*. Further studies are required to determine whether intervening against the systemic and vascular inflammation would control atherogenesis in the presence of active periodontal diseases.

## Materials and methods

### Mice and induction of periodontitis

Male *ApoE*^−/−^ mice on C57BL/6 background (Jackson Laboratory, Bar Harbor, ME) of 8 weeks of age were fed a high-fat diet (HFD) (#D12079B, Research Diets, New Brunswick, NJ) for 11 weeks (*n* = 15). One day after starting the HFD, the *ApoE*^*−/−*^ mice were divided into three groups (*n* = 5 per group): (1) control mice receiving only anesthesia during surgical procedure; (2) mice with subgingival ligature placement with 6-0 silk suture at upper second molars; and (3) mice ligature placement in conjunction with *P.g*. LPS (#tlrl-pglps, InvivoGen, San Diego, CA; 20 μg dissolved in 1 μL of endotoxin-free water) injection at the disto-palatal papilla of both second molars twice a week for 9 weeks.^32^ Ligature was placed under general anesthesia using ketamine/xylazine (100 mg per kg and 5 mg per kg, respectively) as described previously.^34^ For *P.g*. LPS injection (1 µL per injection site), we utilized a 10 μL Hamilton syringe with a 33 gauge needle (Hamilton Company, Reno, NV) under general anesthesia with 2.5% isoflurane administered through a nose cone. All experiments were performed according to the approved institutional guidelines from the Chancellor’s Animal Research Committee (ARC #2016-110).

### Tissue collection and analysis

Whole blood was collected from mice by cardiac puncture under general anesthesia with isoflurane (Abbott Laboratories, Lake Bluff, IL). The mice were then perfused and fixed with 4% paraformaldehyde in phosphate-buffered saline (PBS) via the left ventricle for 5 min. After the perfusion the entire aorta was removed and stained with Oil Red O (Sigma-Aldrich, St. Louis, MO) as previously described.^43^ The atherosclerotic lesion size was determined by ImageJ software (NIH) after Oil Red O staining. Six specimens per group were chosen under randomized and blind condition and quantified. The percentage of plaque area was calculated by dividing the stained area by the total lumen area of the cross-sectional area. The maxillae of the mice were excised and fixed with 4% paraformaldehyde in PBS, pH 7.4, at 4 °C overnight and stored in 70% ethanol solution for micro-computed tomography (μCT) analysis.

### Micro-computed tomography (μCT) analysis

The fixed maxillae were subjected to μCT scanning (Skyscan1275, Bruker-microCT, Kontich, Belgium) using a voxel size of 20 μm^3^ and a 0.5 mm aluminum filter. Two-dimensional slices from each maxilla were combined using NRecon and CTAn/CTVol programs (Bruker) to form a three-dimensional reconstruction. The level of bone resorption was calculated as the distance from the palatal and mesiobuccal CEJ to the ABC by the first author (J.S.S.). The reading was confirmed in a blinded-manner by another author (R.H.K.).

### Histological and immunofluorescence analysis

After μCT scanning, the maxillae were decalcified with 5% EDTA and 4% sucrose in PBS (pH 7.4). Decalcification continued for 3 weeks at 4 °C. The decalcification solution was changed daily. Decalcified maxillae and sectioned aorta were sent to the UCLA Translational Procurement Core Laboratory (TPCL) and processed for paraffin embedding. Blocks were sectioned at 5-μm intervals using a Microtome and slides were dewaxed in xylene. For tartrate-resistant acid phosphatase (TRAP) staining, the sections were stained using an acid phosphatase kit (378A; Sigma-Aldrich) and then, counterstained with hematoxylin. The digital images of the histochemical stained section were obtained using the microscope (DP72, Olympus, Tokyo, Japan).

For immunohistochemical analysis, mouse paraffin-embedded aortic curves were incubated with primary antibodies, PECAM-1 (Santa Cruz Biotechnology, Paso Robles, CA), F4/80 (Abcam, Cambridge, MA), ICAM-1 (BioLegend, San Diego, CA), NOTCH-3 (Abcam), SNAI-1 (Abcam), SM22α (Abcam), and VCAM-1 (Abcam), followed by fluorometric detection with Alexa Fluor 488- or Alexa Fluor 594-conjugated secondary antibodies (Thermo Fisher Scientific, Canoga Park, CA). Sequentially, the sections were mounted on slides with VECTASHIELD anti-fade mounting medium with DAPI (H1200, Vector Laboratories, Burlingame, CA). Slides were investigated with Fluoview FV200i confocal fluorescent microscope (Olympus).

### Serum lipid and cytokine measurements

Levels of total cholesterol, triglycerides, high density lipoprotein (HDL), and non-HDL were measured using enzymatic assay kits in the UCLA Cardiovascular Core Facility.^44^ The serum level of TNF-α, IL-1β, IL-6, and CRP were measured by ELISA (Thermo Fisher Scientific and Sigma-Aldrich). All samples were run in triplicate.

### Cell culture and reagents

Human umbilical vein endothelial cells (HUVECs; Lonza, Basel, Switzerland) were cultured in endothelial basal medium-2 containing EGM-2 SingleQuot Kit (Lonza). The human monocytic leukemia cell line (THP-1) was purchased from ATCC (Manassas, VA) and cultured in RPMI1640 medium containing 10% fetal bovine serum (FBS, Thermo Fisher Scientific) and 1% penicillin/streptomycin. The medium was renewed every 48 h. Cells were cultured at 37 °C and in CO_2_ air atmosphere with a humidity of 5% (v/v). 100 nmol·L^−1^ Phorbol 12-myristate 13-acetate (PMA; Sigma-Aldrich) was treated to differentiate THP-1 cells into macrophages, and untreated THP-1 cells were used as monocytes.

### Induction of EndMT

To study the effects of secreted protein from monocytes or macrophages on the EndMT, HUVECs were co-cultured with monocytes or macrophages differentiated from THP-1 using the Transwell® system (Corning, Corning, NY) for 2 days. To investigate the effects of *P.g*. LPS, TNF-α or transforming growth factor (TGF)-β1/2 on the EndMT of HUVECs, we also exposed HUVECs to *P.g*. LPS (20 µg·mL^−1^), TNF-α (10 ng·mL^−1^), TGF-β1 (10 ng·mL^−1^), or TGF-β2 (10 ng·mL^−1^) for 2 days. The phenotypic changes of HUVECs were analyzed.

### Quantitative real-time polymerase chain reaction

Total RNA from EndMT-undergoing HUVECs was extracted using Trizol-based methods (Thermo Fisher Scientific) and reverse-transcribed using SuperScript® III Reverse Transcriptase Synthesis Kit (Thermo Fisher Scientific). Subsequently, qRT-PCR was performed using PowerUp™ SYBR Green Master Mix (Thermo Fisher Scientific) according to manufacturer’s protocol. The sequences of the primers used for RT-qPCR are described in Table 1. β-Actin served as control and the fold induction was calculated using the comparative ΔCt method and are presented as relative transcript levels (2^−ΔΔCt^).

**Table 1:** Primers for quantitative reverse transcription-polymerase chain reaction (qRT-PCR)

Genes	Forward primer 5′-3′	Reverse primer 5′-3′
*CD31*	GCA ACA CAG TCC AGA TAG TCG T	GAC CTC AAA CTG GGC ATC AT
*FSP1*	GCT CAA CAA GTC AGA ACT AAA GGA G	GCA GCT TCA TCT GTC CTT TTC
*VE-Cad*	AAG CCT CTG ATT GGC ATA GT	CTG GCC CTT GTC ACT GGT
*SLUG*	TGG TTG CTT CAA GGA CAC AT	GCA AAT GCT CTG TTG CAG TG
*GATA4*	GGA AGC CCA AGA ACC TGA AT	GTT GCT GGA GTT GCT GGA A
*TWIST1*	AGA AGT CTG CGG GCT GTG	TCT GCA GCT CCT CGT AAG ACT
*TNF-α*	GCT GCT CAC CTC ATT GGA G	CCA GGA GAG AAT TGT TGC TCA
*IL-1β*	AAT CTG TAC CTG TCC TGC GTG TT	TGG GTA ATT TTT GGG ATC TAC ACT CT
*IL-6*	CTT TTG GAG TTT GAG GTA TAC CTA G	GCT GCG CAG AAT GAG ATG AGT TGT C
*β-actin*	CCA ACC GCG AGA AGA TGA	CCA GAG GCG TAC AGG GAT AG

### Immunoblotting

Total protein from EndMT-undergoing HUVECs was extracted and size-fractioned by SDS-polyacrylamide gel electrophoresis and transferred to nitrocellulose membranes. After blocking with 5% skim milk in PBS with 0.1% Triton-X100, immunodetection was carried out using specific primary antibodies: anti-p-p65 (Cell Signaling, Danvers, MA), anti-p65 (Santa Cruz Biotechnology), anti-p-Smad2 (Cell Signaling), anti-Smad2 (Cell Signaling), anti-p-p38 (Cell Signaling), anti-p38 (Cell Signaling), anti-p-Erk (Cell Signaling), anti-Erk (Cell Signaling), anti-TWIST (Santa Cruz Biotechnology), anti-FSP-1 (Abcam), anti-CD31 (Abcam), anti-VE-Cadherin (Abcam), anti-α-SMA (Sigma-Aldrich), anti-E-Selectin (Santa Cruz Biotechnology). Glyceraldehyde 3-phosphate dehydrogenase (GAPDH) (Santa Cruz Biotechnology); was used as loading control. Thereafter, blots were incubated with HRP-labeled respective (anti-mouse or anti-rabbit) secondary antibodies (Santa Cruz Biotechnology), washed and processed with Clarity™ Western ECL Substrate (Thermo Fisher Scientific). Signal was detected using ChemiDoc™ Touch image analyzer (Bio-Rad Laboratories).

### Immunocytochemistry

Immunostaining was performed in 4-well chamber slide. After fixing with 4% paraformaldehyde for 10 min and washing with PBS, blocking solution (5% bovine serum albumin in PBS with 0.1% Triton-X100) was applied for 1 h. Primary antibodies (anti-CD31: Abcam, anti-FSP1: Abcam, anti-VE-Cadherin: Abcam and anti-α-SMA: Sigma-Aldrich) were applied overnight at 4 °C. Cells were washed with PBS, and corresponding fluorescence-tagged secondary antibodies were applied for 1 h at room temperature. After washing, the cells were mounted using Vectashield mounting medium with DAPI. Immunostaining was observed under Olympus Fluoview FV200i confocal fluorescent microscope (Olympus).

### Enzyme‐linked immunosorbent assay (ELISA)

Levels of IL-6, IL-1β, and TNF-α in supernatants from THP-1 derived cells were measured by ELISA using Ready‐SET‐go kits (Thermo Fisher Scientific) according to manufacturer’s protocol. The color reaction was stopped with the addition of Stop solution (BioLegend), and absorbance was read immediately using a plate reader at 450 nm (Bio-Rad Laboratories). The standard curve was calculated by plotting the standards against the absorbance values, and the cytokine levels were measured in pg/ml.

### Cell adhesion assay

Labeled THP-1 cells with Calcein-AM (Sigma-Aldrich) were co-cultured on monolayers of HUVECs in chamber slides, exposed to either culture medium only or *P.g*. LPS for 1 h. Thirty minutes after the co-culture, the culture dish was washed thoroughly, and the adhered THP-1 cells were determined using Olympus Fluoview FV200i confocal fluorescence microscope (Olympus).

### Analysis of secretory pro-inflammatory cytokines and chemokines in vitro

The cytokine and chemokine expression profiles of the monocytes and macrophages were determined using a Human Cytokine Antibody Array kit (RayBiotech Inc., Norcross, GA).

### Statistical analyses

All graphs were created using GraphPad Prism software, and statistical analyses were calculated using GraphPad Prism 5. For multiple comparisons, 1-way ANOVA with Newman–Keuls test was used. A *P*-value of less than 0.05 was considered significant. All results from in vitro were confirmed by at least 3 independent experiments. Error bars represent mean ± SEM.

## Supplementary information

Periodontitis-induced systemic inflammation exacerbates atherosclerosis partly via endothelial-mesenchymal transition in mice
