# Supplementary material for: Periodontitis-induced systemic inflammation exacerbates atherosclerosis partly via endothelial–mesenchymal transition in mice
Source: Int J Oral Sci. 2019 Jul 1;11(3):21. doi: 10.1038/s41368-019-0054-1 (PMC6802639; doi:10.1038/s41368-019-0054-1)
Supplement: Supplementary file 1 — Periodontitis-induced systemic inflammation exacerbates atherosclerosis partly via endothelial-mesenchymal transition in mice [file 41368_2019_54_MOESM1_ESM.docx]

**SUPPLEMENTARY MATERIAL**

**Periodontitis-induced systemic inflammation exacerbates atherosclerosis partly *via* endothelial-mesenchymal transition in mice**

Jin Sook Suh^1^, Sol Kim^1^, Kristina I. Boström^2^, C-Y Wang^1, 3, 4^, Reuben H Kim^1, 3^, and No-Hee Park^1, 2, 3^

^1^The Shapiro Family Laboratory of Viral Oncology and Aging Research, UCLA School of Dentistry, 10833 Le Conte Ave, Los Angeles, CA, USA

^2^Department of Medicine, David Geffen School of Medicine at UCLA, 10833 Le Conte Ave, Los Angeles, CA, USA

^3^UCLA Jonsson Comprehensive Cancer Center, 10833 Le Conte Ave, Los Angeles, CA, USA

^4^Department of Bioengineering, UCLA Samueli School of Engineering, Los Angeles, CA, USA

Running title: Periodontitis induces Atherosclerosis *via* Inflammation

Correspondence: No-Hee Park, DMD, PhD,

43-005 CHS, Box 951668, University of California, Los Angeles, CA 90095-1668

310-825-0339 (voice), nhpark@ucla.edu (email)

**
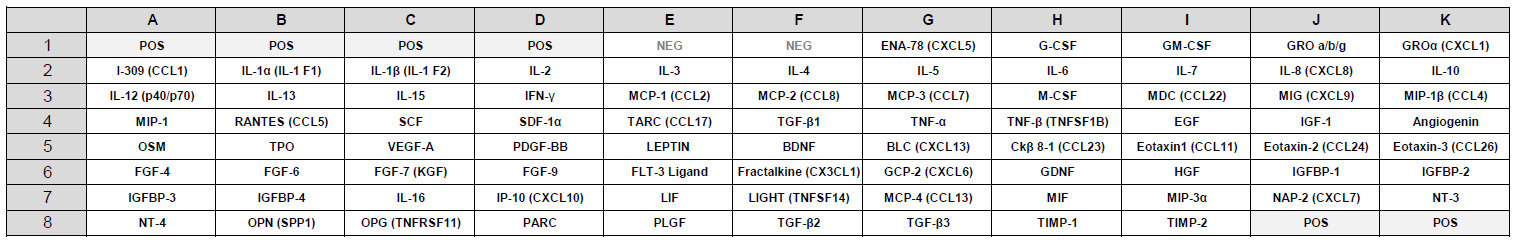
**

**Supplementary Table 1. Cytokine array map**

**
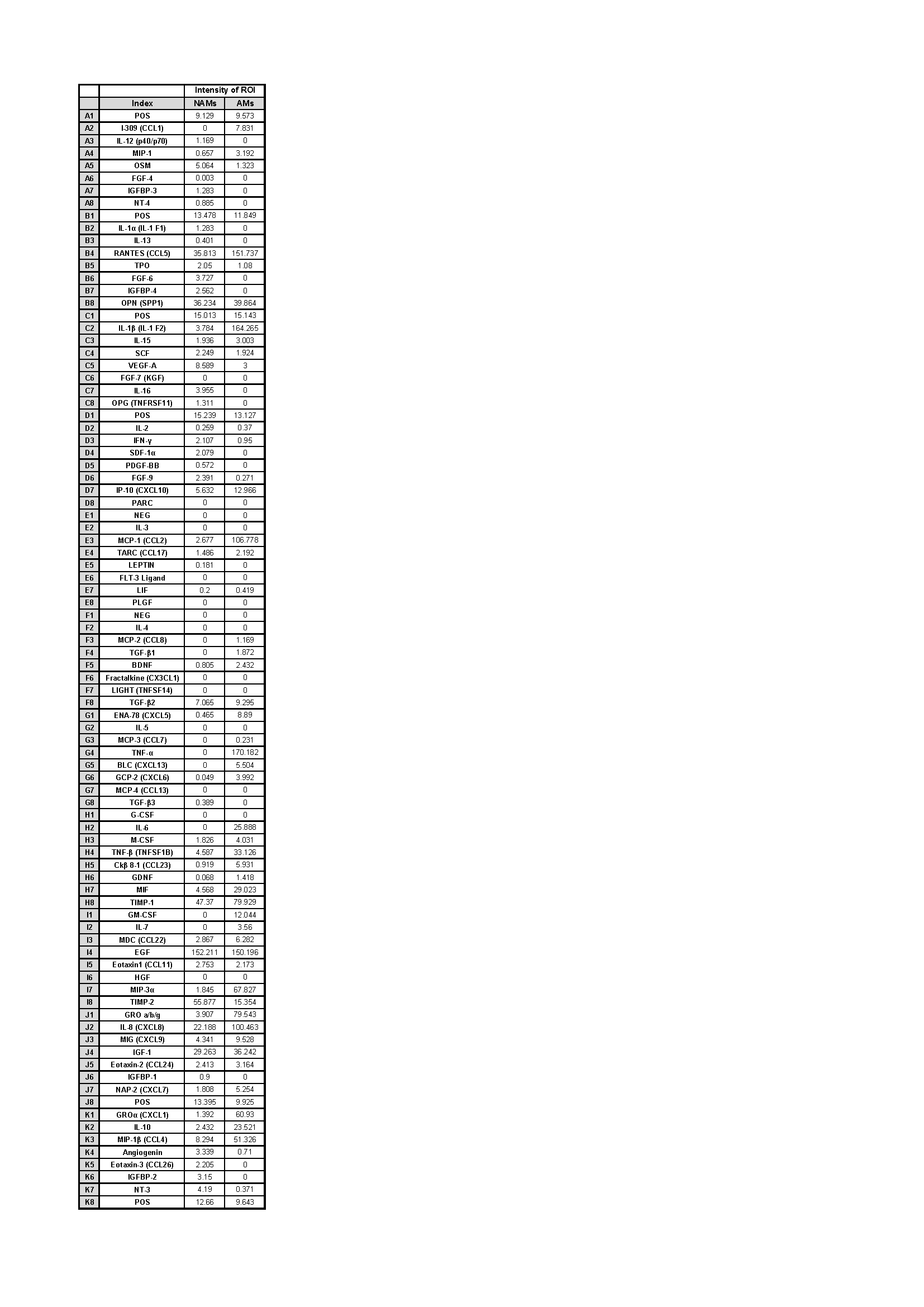
**

**Supplementary Table 2. The raw numerical densitometry data of cytokine array membranes.** The optical density of each detected proteins was calculated using ImageJ software (NIH).

**
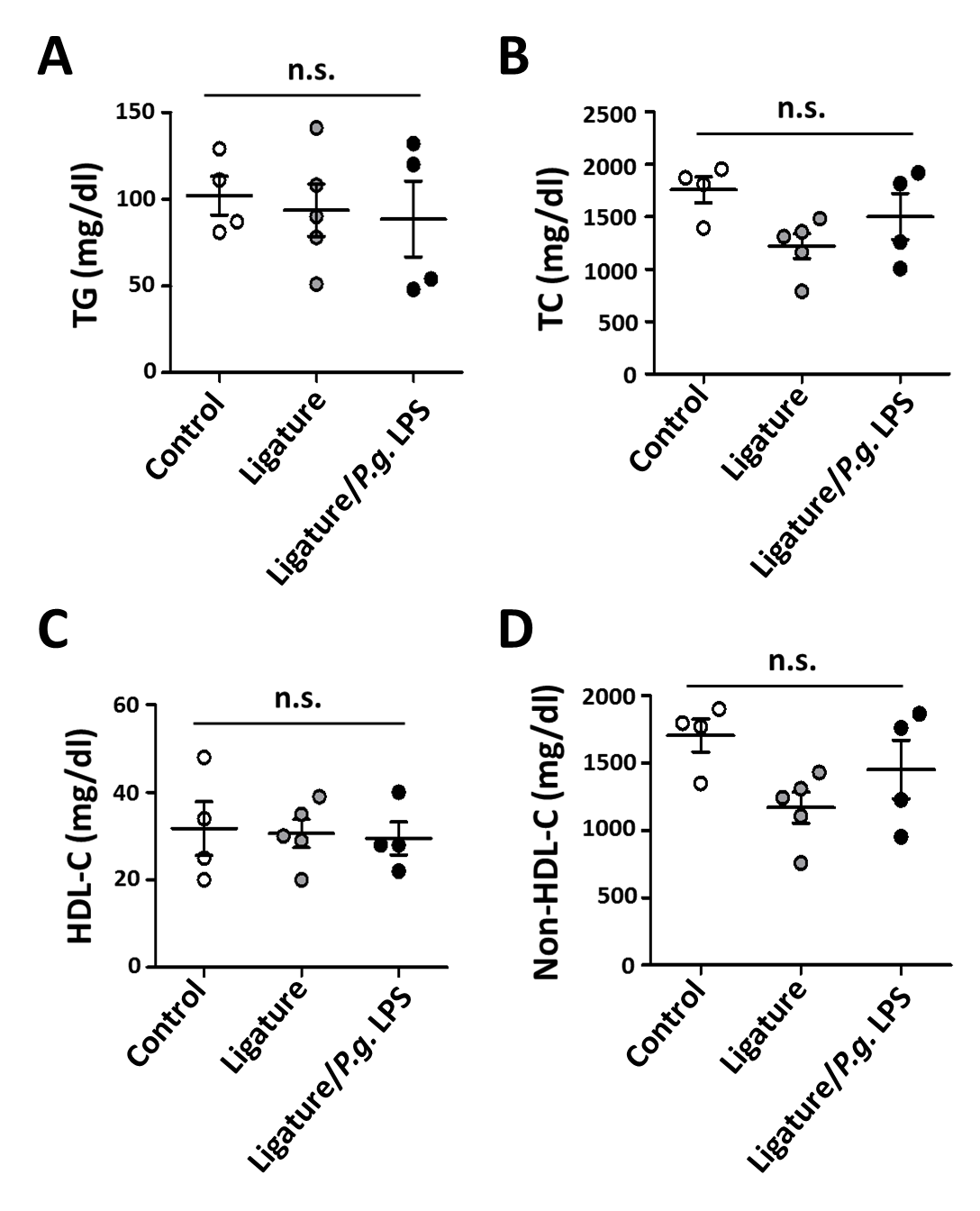
**

**Supplementary Figure 1. Periodontitis induced by Ligature placement or Ligature/*P.g.* LPS didn’t affect to the levels of lipid in mouse serum.** Levels of Serum lipid: (A) triglyceride (TG), (B) total cholesterol (TC), (C) high-density lipoprotein cholesterol (HDL-C), and (D) non-high density lipoprotein cholesterol (Non-HDL-C). n.s.: not significant. **p* < 0.05; ***p* < 0.01; ****p* < 0.001 in one-way ANOVA. Results represent the means ± SD performed in triplicate.

**
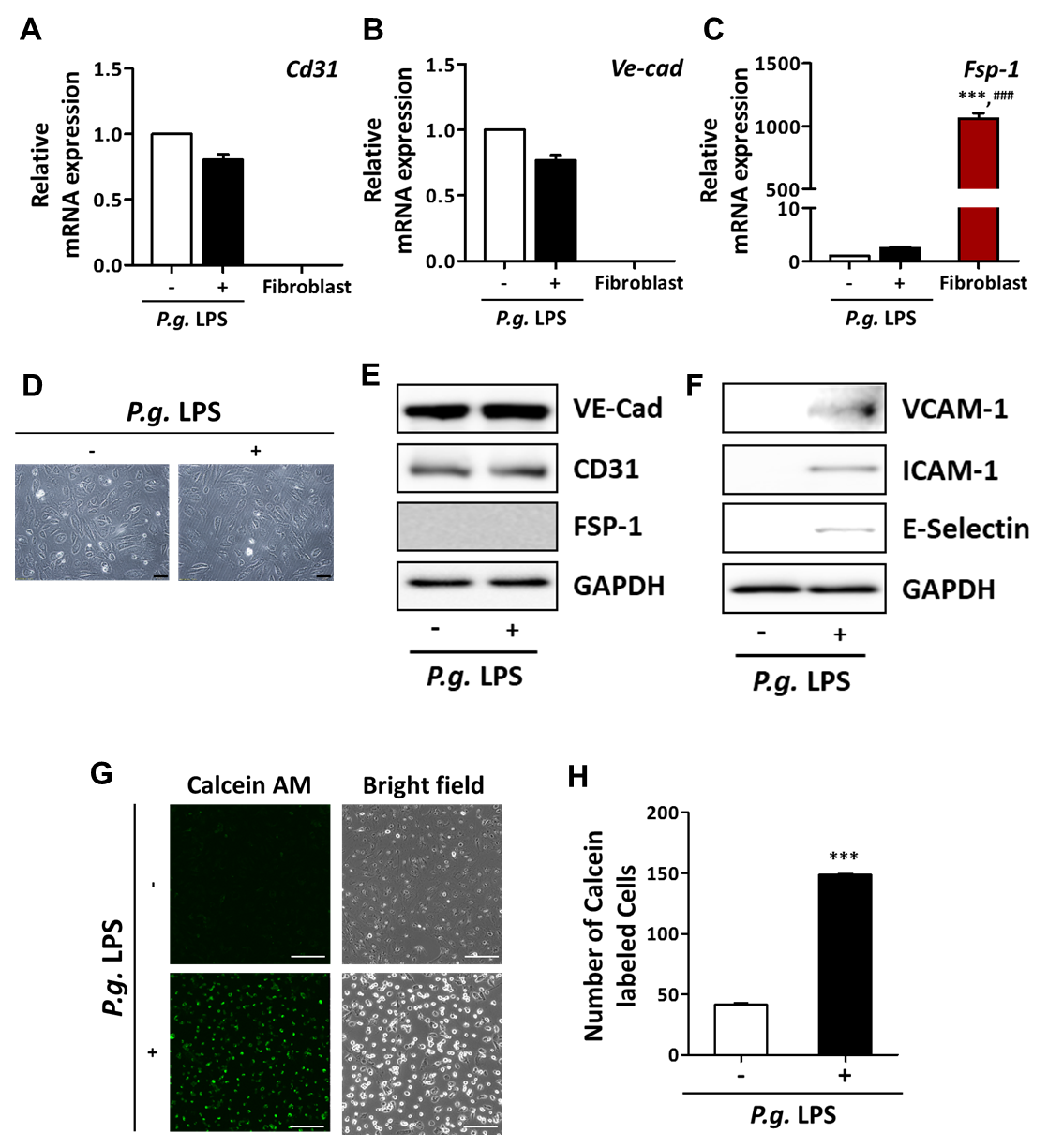
**

**Supplementary Figure 2. *P.g.* LPS failed to induce EndMT of HUVECs, but it enhanced expression of cell-adhesion molecules in endothelial cells, resulting in the adhesion of monocytes to HUVECs.** (A-C) Relative mRNA expressions of VE-Cad, CD31, and FSP-1 in *P.g.* LPS treated HUVECs. Human fibroblast was used as a positive control cells for FSP-1 expression. **p* < 0.05; ***p* < 0.01; ****p* < 0.001 compared with non-treated HUVECs and #*p* < 0.05; ###*p* < 0.001 compared with *P.g.* LPS-treated HUVECs in one-way ANOVA. Results represent the means ± SD performed in triplicate. (D) Phase contrast microscopy of HUVECs grown in basal media or media containing *P.g.* LPS for 2 days. Scale bars: 100μm. (E) Representative images from Western blot experiments performed for the detection of endothelial markers CD31 and VE-cad, a fibrotic marker, FSP-1 in non-treated or *P.g.* LPS-treated HUVECs. *P.g.* LPS failed to express FSP-1 in HUVECs. (F) Representative images from Western blot experiments performed for the detection of adhesion molecules such as VCAM-1, ICAM-1 and E-Selectin in non-treated or *P.g.* LPS-treated HUVECs. *P.g.* LPS failed to alter the expression of these endothelial cell protein markers. (G) The adhesion of fluorescence-tagged THP1 monocytes to HUVECs or HUVECs exposed to *P.g.* LPS. THP1 cells were labeled with Calcein AM and placed on two groups of HUVECs monolayer for 1h. Scale bars: 100μm. (H) Quantification of attached Calcein AM-labeled THP1 cells. ****p* < 0.001 in two-sided Student’s *t*-test.
